# SR-BI associates with ABCG1 and inhibits ABCG1-mediated cholesterol efflux from cells to high-density lipoprotein 3

**DOI:** 10.1186/1476-511X-11-118

**Published:** 2012-09-17

**Authors:** Guohua Song, Chuanlong Zong, Qian Liu, Yanhong Si, Jie Liu, Wei Li, Ping Zhu, Shucun Qin

**Affiliations:** 1 Key Laboratory of Atherosclerosis in Universities of Shandong; Institute of Atherosclerosis, Taishan Medical University, Taian, China; 2 PLA General Hospital, Beijing, China; 3 2# YingSheng E Road, Taian, Shandong, 271000, P.R.China; 4 28# Fuxing Road, Beijing, 100853, P.R.China

## Abstract

**Background:**

The single and combined effects of scavenger receptor-BI (SR-BI), ATP-binding cassette transporter (ABC) A1 and G1 on cholesterol efflux from Chinese Hamster Ovary (CHO) cells were investigated.

**Results:**

When apolipoproteinA-I (apoA-I) was used as an acceptor, ABCA1 overexpression led to an increase in total cholesterol (TC) in medium which is attributable to a 2-fold increase in free cholesterol (FC) content. When high-density lipoprotein 3 (HDL3) was used as an acceptor, SR-BI overexpression not only promoted FC efflux, but also promoted the uptake of cholesteryl ester (CE) into cells, resulting in no TC varieties in medium. Overexpression of ABCG1 increased both the FC and CE levels in medium. However, when apoA-I and HDL3 were both used as acceptors, coexpression of SR-BI has no effect on ABCA1-mediated increased FC and TC accumulation in medium. Interestingly, coexpression of SR-BI with ABCG1 blocked the ABCG1-mediated cholesterol efflux to HDL3, mostly by promoting the reuptake of CE from the medium. Furthermore, co-immunoprecipitation experiments revealed that SR-BI interacted with ABCG1 in BHK cells overexpressing ABCG1 and SR-BI.

**Conclusions:**

We found SR-BI associates with ABCG1 and inhibits ABCG1-mediated cholesterol efflux from cells to HDL3.

## Background

Reverse cholesterol transport (RCT) is assumed to play a critical role in the pathogenesis of atherosclerosis [1]. Cellular cholesterol efflux, by which cholesterol is transported from peripheral cells to high-density lipoprotein (HDL) acceptor molecules, is the first step of RCT [2]. ATP-binding cassette transporter (ABC) A1, G1 and scavenger receptor-BI (SR-BI) are three important mediators of cholesterol efflux. ABCA1 exports cholesterol and phospholipid to lipid-poor apolipoproteins and initiates HDL formation by lipidating apolipoproteinA-I (apoA-I) [3]. However, ABCA1 interacts poorly with HDL2 and HDL3 particles, which constitute the bulk of the plasma HDL. Another two cell surface transporters, ABCG1 and SR-BI, have been described to export cholesterol to phospholipid-containing acceptors, including HDL. SR-BI stimulates the bidirectional flux of cholesterol between cells and lipoproteins [4], while ABCG1 has been shown to promote free cholesterol (FC) efflux from macrophage to HDL and increase the accumulation of cholesteryl ester (CE) in medium as a result of lecithin-cholesterol acyltransferase (LCAT)-mediated cholesterol esterification [5].

It has been reported that mice lacking ABCA1 expression in macrophages developed accelerated atherosclerosis [6]. Conversely, mice with macrophage overexpression of ABCA1 have decreased atherosclerosis [7]. Mice lacking ABCG1 have evidence of lipid accumulation in certain tissue macrophages [8,9]. Furthermore, transplantation of bone marrow from SR-BI deficient (SR-BI −/−) mice into LDL receptor −/− or apoE −/− mice have shown an increase in atherosclerosis [10-12]. Taken together, these findings suggest that all three transporters involving macrophage ABCA1, ABCG1 and SR-BI may exert protective functions in atherosclerosis. In addition, many reports have indicated a synergistic relationship between ABCA1 and ABCG1 in peripheral tissues, where ABCA1 lipidates any lipid-poor/free apoA-I to generate nascent or pre–ß-HDL. These particles in turn may serve as substrates for ABCG1-mediated cholesterol export [13-15]. However, the relationships between SR-BI and the two ABC transporters are not well understood. The current study was undertaken to compare the roles of SR-BI and the two ABC transporters in promoting mass cholesterol efflux and determine a possible interaction between the SR-BI- and ABC transporter-mediated cholesterol efflux pathways in vitro. We found coexpression of SR-BI with ABCG1 blocked the ABCG1-mediated cholesterol efflux to HDL3, mostly by promoting the reuptake of CE from the medium. Furthermore, co-immunoprecipitation experiments revealed that SR-BI interacted with ABCG1 in BHK cells overexpressing ABCG1 and SR-BI, which might play roles in maintaining cellular cholesterol homeostasis.

## Results

### The single effects of SR-BI, ABCA1 and ABCG1 on cellular cholesterol efflux

**Figure 1:**
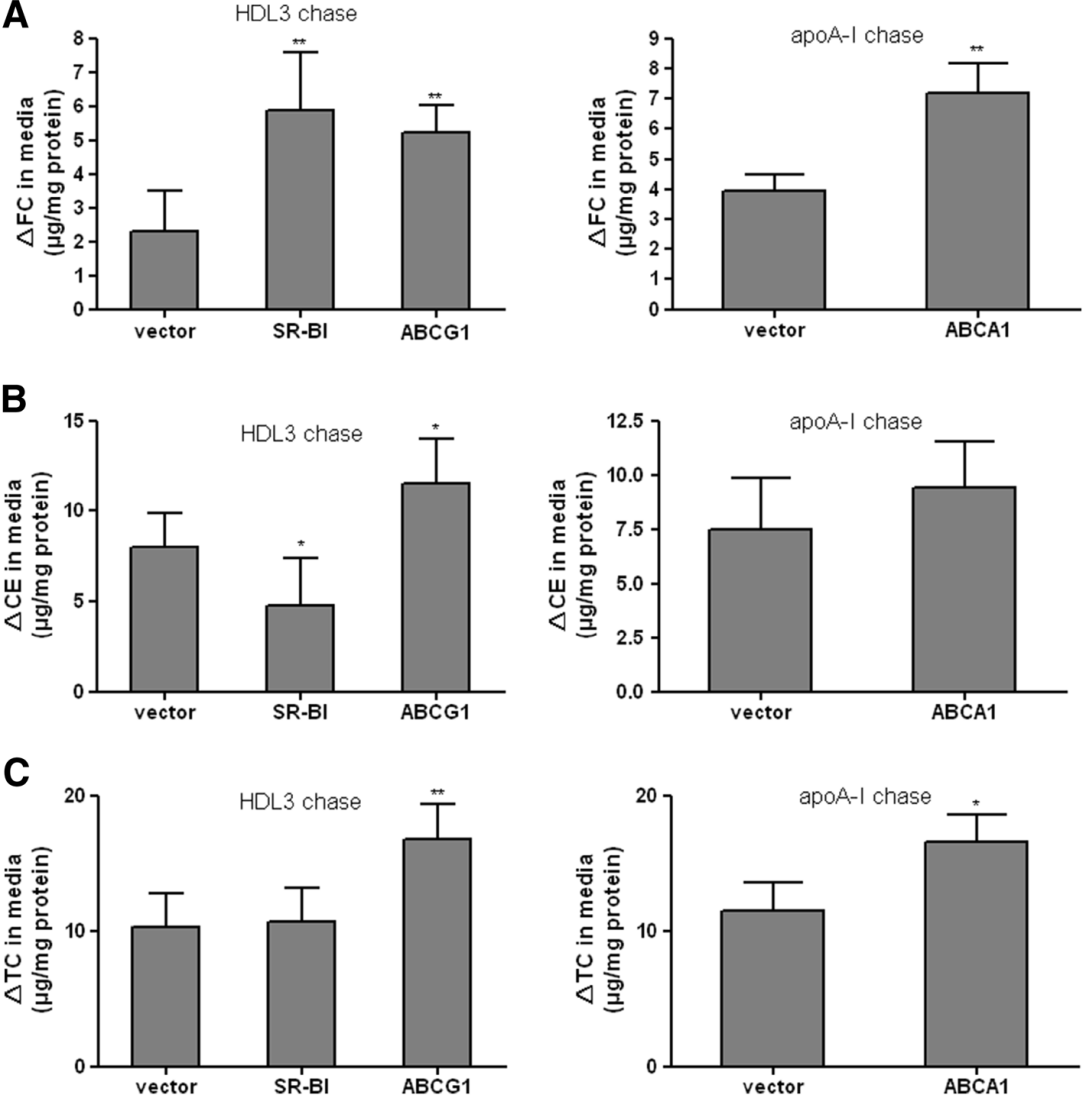
**The single effects of SR-BI, ABCA1 and ABCG1 on cellular cholesterol efflux.** CHO cells were transiently transfected with plasmid constructs expressing SR-BI, ABCA1, ABCG1 or empty vector. 24 h after transfection, the cells were incubated with 150 μg/ml HDL3 (HDL3 chase) or 10 μg/ml apoA-I (apoA-I chase) for 24 h in Opti-MEM I Medium. Cholesterol efflux was determined by measuring the increased free cholesterol (FC, A), cholesteryl ester (CE, B), and total cholesterol (TC, C) in medium. Values are means ± SD of three independent experiments. *p < 0.05, **p < 0.01.

It has been reported that lipid-free apoA-I is the lipid acceptor of ABCA1-mediated cholesterol efflux [16-18], while SR-BI [19] and ABCG1 [8,20] were identified as being mediators of cholesterol efflux to mature HDL, but not to lipid-free apoA-I in vitro. Therefore, we used apoA-I as lipid acceptors in ABCA1 transfected cells and HDL3 as lipid acceptors in SR-BI or ABCG1 transfected cells. As shown in Figure 1, SR-BI overexpression not only increased the accumulation of FC (Figure 1A), but also decreased the accumulation of CE in medium (Figure 1B), resulting in no total cholesterol (TC) varieties in medium (Figure 1C). These data gave us a clue that SR-BI overexpression might not only promote FC efflux, but also promote the uptake of CE into cells. In addition, ABCA1 overexpression led to an increase in TC in medium (Figure 1C), in large part attributable to a 2-fold increase in FC content (Figure 1A), while overexpression of ABCG1 increased both the FC (Figure 1A) and CE (Figure 1B) levels in medium. 

### The effects of SR-BI on ABCA1-mediated cellular cholesterol efflux

**Figure 2:**
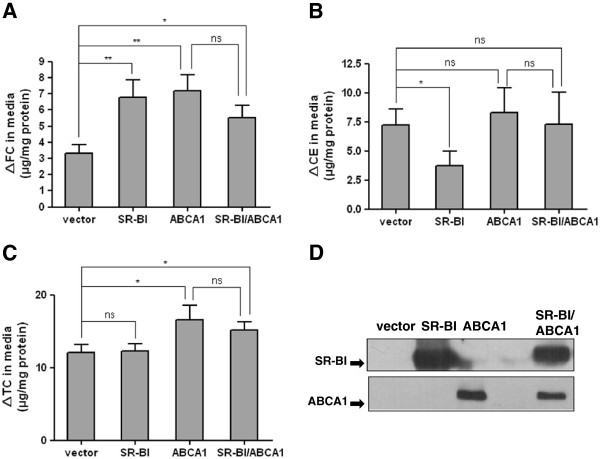
**SR-BI does not affect ABCA1-mediated cellular cholesterol efflux.** CHO cells were transiently transfected with SR-BI alone, ABCA1 alone, and SR-BI plus ABCA1 (co-transfection). 24 h after transfection, cholesterol efflux was initiated by the addition of HDL3 (150 μg protein/ml) and apoA-I (10 μg/ml) to medium for 24 h. The increased FC (**A**), CE (**B**) and TC (**C**) mass in medium were determined. Values are means ± SD of three independent experiments. * p < 0.05, ** p < 0.01. **D** shows the western blots of SR-BI and ABCA1 protein in vector transfected, SR-BI transfected, ABCA1 transfected and cotransfected cells.

Because ABCA1 and ABCG1 act in a cooperative fashion to promote cellular cholesterol efflux [13], we next determined whether SR-BI might facilitate ABCA1-mediated cellular cholesterol efflux. Thus, we coexpressed SR-BI and ABCA1 in CHO cells and determined the cholesterol efflux to apoAI and HDL3 (apoA-I and HDL3 were both used as acceptors). As shown in Figure 2, coexpression of SR-BI with ABCA1 has no effect on ABCA1-mediated increased FC (Figure 2A) and TC (Figure 2B) accumulation in medium. Also, the CE levels in medium were not changed in SR-BI and ABCA1 cotransfected cells compared with cells overexpressing ABCA1 alone (Figure 2C). These data indicated that SR-BI overexpression has no effect on ABCA1-mediated cellular cholesterol efflux to apoAI and HDL3 in vitro. Moreover, the expression levels of ABCA1 were similar in cells transfected with ABCA1 alone or ABCA1 plus SR-BI (Figure 2D), which indicates that our findings were not attributable to a difference in ABCA1 expression levels. 

### The effects of SR-BI on ABCG1-mediated cellular cholesterol efflux

**Figure 3:**
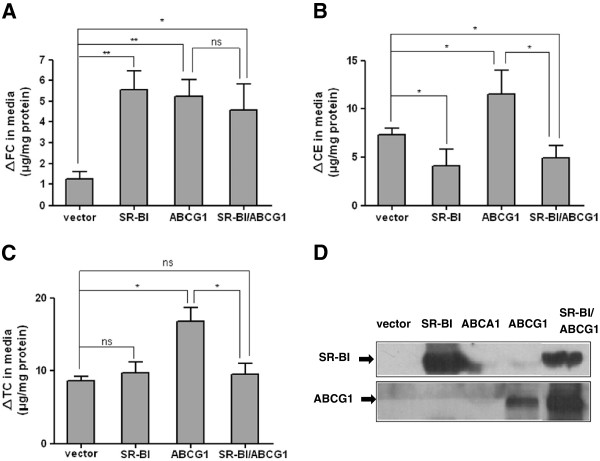
**SR-BI inhibits ABCG1-mediated cellular cholesterol efflux.** CHO cells were transiently transfected with SR-BI alone, ABCG1 alone, and SR-BI plus ABCG1 (co-transfection). 24 h after transfection, cholesterol efflux was initiated by the addition of HDL3 (150 μg protein/ml) to medium for 24 h. The increased FC (**A**), CE (**B**) and TC (**C**) mass in medium were determined. Values are means ± SD of three independent experiments. * p < 0.05, ** p < 0.01. **D** shows the western blots of SR-BI and ABCG1 protein in vector transfected, SR-BI transfected, ABCG1 transfected and cotransfected cells.

To evaluate the role of SR-BI on ABCG1-mediated cholesterol efflux, we coexpressed SR-BI and ABCG1 in CHO cells and determined the cholesterol efflux to HDL3. As shown in Figure 3, coexpression of SR-BI with ABCG1 blocked the ABCG1-mediated cholesterol efflux to HDL3, and it seems that this effect of SR-BI was mostly achieved by promoting the reuptake of CE from the medium. This was similar to previous findings, in which it was indicated SR-BI inhibits ABCG1-stimulated CE accumulation in medium in an LCAT-dependent manner by using HEK293 cell model [21]. 

### SR-BI co-immunoprecipitated with ABCG1 in BHK cells overexpressing SR-BI and ABCG1

**Figure 4:**
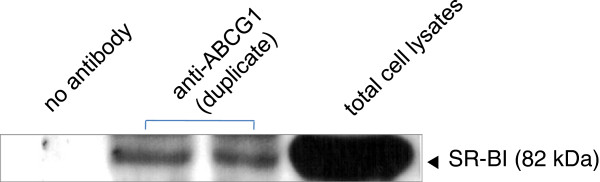
**Co-immunoprecipitation of SR-BI with ABCG1 in BHK cells overexpressing SR-BI and ABCG1.** The cell lysates from the transfected BHK cells were immunoprecipitated with anti-ABCG1 antibody. The immunoprecipitates were then analyzed by western blot with antibody against SR-BI.

Co-immunoprecipitation experiments with cell lysates from SR-BI and ABCG1 co-transfected BHK cells were performed to confirm whether SR-BI and ABCG1 were present in the same complex. The immunoprecipitates were then analyzed by western blot with antibodies against SR-BI. As shown in Figure 4, SR-BI were detected in the anti-ABCG1 immunoprecipitates from the cell lysates of transfected BHK cells. These results suggested that SR-BI associates with ABCG1 in BHK cells overexpressing SR-BI and ABCG1.

## Discussion

The purpose of the present study was to compare the roles of SR-BI, ABCA1 and ABCG1 singly or together in mediating cholesterol efflux from cells in vitro. We found that SR-BI associates with ABCG1 and inhibits ABCG1-mediated cholesterol efflux from cells to HDL3, and that overexpression of SR-BI has no effect on ABCA1-mediated cellular cholesterol efflux to apoAI and HDL3 in vitro.

It has been reported that ABCA1 exports cholesterol and phospholipid to lipid-poor apolipoproteins, and interacts poorly with HDL2 and HDL3 particles [3]. ABCG1 and SR-BI have been described to export cholesterol to phospholipid-containing acceptors, including HDL [4]. In our study, overexpression of ABCA1 promoted the efflux of FC from cells to apoA-I and overexpression of ABCG1 not only promoted the FC efflux to HDL3, but also increased the CE levels in medium. A previous study showed that ABCG1 increases the accumulation of CE in medium as a result of LCAT-mediated cholesterol esterification [5], so it is possible that the increase in CE induced by ABCG1 in our study might be attributable to the active LCAT, which is retained in the HDL3 particles. Previous findings have shown that ABCA1 and ABCG1 synergize to mediate cellular cholesterol export to lipoproteins [13], so we initially hypothesized that SR-BI and ABCA1 might also act synergistically on cholesterol efflux, because the nascent HDL particles formed from the ABCA1-mediated cholesterol efflux to apoA-I might serve as an acceptor for SR-BI-mediated cholesterol efflux. To assess the hypothesis, we performed cotransfection with SR-BI and ABCA1 cDNAs in CHO cells and then treated the cells with cholesterol acceptor apoA-I and HDL3. Out of our expectation, we found ABCA1 promoted the efflux of FC from cells, but SR-BI has no effect on ABCA1-stimulated increased FC accumulation in medium. Furthermore, we co-transfected SR-BI and ABCG1 cDNAs into CHO cells and found an interesting phenomenon that SR-BI inhibited ABCG1-mediated CE accumulation in medium. This was similar to a previous finding, in which it was shown SR-BI promotes cellular reuptake of the CE that initially undergoes ABCG1-mediated cholesterol efflux to HDL3 containing active LCAT [21]. In this context, we thought it is possible that SR-BI and ABCG1 might talk to each other in cells. Therefore, we performed co-immunoprecipitation experiments with cell lysates from SR-BI and ABCG1 co-transfected BHK cells to confirm whether SR-BI and ABCG1 were present in the same complex. In our expectation, we found SR-BI associates with ABCG1 in transfected BHK cells. Based on our findings, it is possible that in our experimental conditions, SR-BI interacted with ABCG1 and therefore inhibited ABCG1-mediated cholesterol efflux.

The futile cycles created by the competing role of SR-BI and ABCG1 can have physiological relevance in tissues expressing ABC transporters, such as macrophages or liver [16,22]. But future studies need to be performed to confirm the effect of SR-BI on ABCA1- or ABCG1-mediated cholesterol efflux to apoA-I or HDL in macrophages and hepatocytes under basal physiological conditions because the expression levels of SR-BI and ABC transporters were different among different cell types. In addition, we performed experiments in nonploarized cells, it is possible that in polarized cells, such as hepatocytes, SR-BI and ABC transporters may be expressed on different sides of the cell and the futile cycle created by the joint overexpression of SR-BI and ABCG1 in CHO cells and BHK cells might be avoided.

Besides, we also found SR-BI promoted FC efflux to HDL3, but this was counteracted by an increased uptake of CE into cells and thus no significant change in TC in medium. This was similar to previous findings that SR-BI promoted a bidirectional flux of isotopic cholesterol between cells and lipoproteins [23,24], but our study extends earlier findings suggesting that SR-BI singly does not play important roles in promoting net cholesterol efflux to HDL3. Although SR-BI did not promote net cholesterol efflux, it is possible that the cholesterol changes mediated by SR-BI may have effects on cellular cholesterol homeostasis, because CE entering the cells would not play regulatory roles corresponding to that resulting from the efflux of FC.

## Conclusions

Our data suggested SR-BI associates with ABCG1 and appear to inhibit ABCG1-mediated CE accumulation in medium, but does not affect ABCA1-stimulated cholesterol efflux from cells to apoA-I or HDL3 in vitro, which might play roles in maintaining cellular cholesterol homeostasis. Further studies on the interactions between endogenous SR-BI and ABC transporters in promoting cholesterol efflux in vivo need to be conducted.

## Methods

### Isolation of HDL from plasma

HDL3 fractions were isolated from normolipidemic human plasma by sequential ultracentrifugation at a density of 1.210 g/ml and dialyzed against PBS containing 1 mM EDTA [25]. The integrity of the isolated HDL3 was checked by 0.8% agarose gel electrophoresis.

### Cell culture and transfection

Chinese hamster ovary (CHO) cells purchased from ATCC were cultured in F-12 medium (Hyclone) supplemented with 10% (v/v) fetal bovine serum (FBS, Gibco) and 2 mM _L_-glutamine. Cells grown to 80-90% confluence were transiently transfected with similar amounts of control empty vector (pcDNA3.1^+^), murine SR-BI, ABCA1 or ABCG1 cDNA alone combined with empty vector (1:1), or both SR-BI and ABCA1 cDNA (1:1), or both SR-BI and ABCG1 cDNA (1:1) using Lipofectamine 2000 (Invitrogen) according to the manufacturer`s instructions. BHK-G (BHK cells stably expressing mifepristone inducible ABCG1) cells were kindly provided by Dr. Xian-cheng Jiang, SUNY Downstate Medical Center, USA. 10 nM mifepristone in 1% fatty acid-free bovine serum albumin was used for 18–20 hr to induce ABCG1 expression. Then BHK-G cells were transiently transfected with SR-BI using Lipofectamine 2000 and incubated for 24–36 hr prior to performing the further experiments. For all experiments, cells cultured for 4–10 passages were used.

### Western blot analysis

Transfected cells were washed with phosphate-buffered saline (PBS) and lysed in lysis buffer containing 50 mM Tris, pH 7.5, 1.0 mM EDTA, 150 mM NaCl, 0.1% SDS, 1% TritonX-100, 1% Sodium deoxycholate, and 1 × proteinase inhibitor cocktail (Invitrogen). After heating at 95°C for 5 min, cell-lysate samples of equal protein concentration were subjected to SDS-PAGE and transferred onto polyvinylidene fluoride (PVDF) membranes (Millipore, USA) by electroblotting. ABCA1, ABCG1 and SR-BI proteins were detected using anti-ABCA1 polyclonal antibody (1:200 dilution, Santa Cruz Biotechnology), anti-ABCG1 polyclonal antibody (1:300, Santa Cruz Biotechnology), or anti-SR-BI monoclonal antibody (1:2000, Novus Biological Inc.). Primary antibodies were detected using a peroxidase-conjugated anti-mouse, anti-goat or anti-rabbit antibody and revealed by ECL Substrate (Pierce).

### Net cholesterol efflux assays

Cholesterol efflux assays were carried out as described with modifications [5]. In brief, 24 hours after transfection, the cells were incubated for 24 h in Opti-MEM serum-free medium (Invitrogen) in the presence or absence of HDL3 (150 μg protein/ml) and/or apoA-I (10 μg/ml, Sigma). After the incubation, the mass of cholesterol in medium was determined by using Cholesterol/Cholesteryl Ester Quantitation Kit (Biovision) according to the manufacturer`s instructions. Cells were lysed and the protein content was determined by the BCA method.

### Co-immunoprecipitation and Western blot

BHK cells transfected with ABCG1 and SR-BI were used for immunoprecipitation experiments. Immunoprecipitation of SR-BI and ABCG1 protein was performed by using the Protein G Immunoprecipitation Kit (Roche) according to the manufacturer’s instructions. The immunoprecipitated proteins were then subjected to western blotting to analyze the target antigens in complex mixtures of proteins.

### Statistical analysis

Statistical analysis was performed by one-way analysis of variance (ANOVA) test with the GraphPad Prism programme ver.4.0. Results are expressed as means ± SD. P values less than 0.05 were considered significant.

## Competing interests

The authors declare that there are no conflicts of interest in our manuscript.

## Authors’ contributions

GS carried out the study design, data collection and analysis, and drafted the manuscript. CZ participated in the cell-based experiments and review of the manuscript. QL performed the co-immunoprecipitation experiments. YS and JL conducted the isolation of HDL. WL participated in editing the manuscript. SQ and PZ was responsible for the study design, the funding, the data analysis, and the manuscript draft. All authors read and approved the final manuscript.
